# Progress in Sol-Gel Technology for the Coatings of Fabrics

**DOI:** 10.3390/ma13081838

**Published:** 2020-04-14

**Authors:** Aravin Prince Periyasamy, Mohanapriya Venkataraman, Dana Kremenakova, Jiri Militky, Yan Zhou

**Affiliations:** 1 Department of Material Engineering, Faculty of Textile Engineering, Technical University of Liberec, Studentska 2, 46117 Liberec, Czech Republic; mohanapriya.venkataraman@tul.cz (M.V.); dana.kremenakova@tul.cz (D.K.); jiri.militky@tul.cz (J.M.); 2 College of Textile and Clothing Engineering, Soochow University, 199 Renai Road, Suzhou 215123, China; yanzhao@suda.edu.cn

**Keywords:** functional finishing, sol-gel coating, surface modification, coating, protective properties, oil/water separation, fabric finishing

## Abstract

The commercial availability of inorganic/organic precursors for sol-gel formulations is very high and increases day by day. In textile applications, the precursor-synthesized sol-gels along with functional chemicals can be deposited onto textile fabrics in one step by rolling, padding, dip-coating, spraying or spin coating. By using this technology, it is possible to provide fabrics with functional/multi-functional characteristics including flame retardant, anti-mosquito, water- repellent, oil-repellent, anti-bacterial, anti-wrinkle, ultraviolet (UV) protection and self-cleaning properties. These surface properties are discussed, describing the history, basic chemistry, factors affecting the sol-gel synthesis, progress in sol-gel technology along with various parameters controlling sol-gel technology. Additionally, this review deals with the recent progress of sol-gel technology in textiles in addressing fabric finishing, water repellent textiles, oil/water separation, flame retardant, UV protection and self-cleaning, self-sterilizing, wrinkle resistance, heat storage, photochromic and thermochromic color changes and the improvement of the durability and wear resistance properties.

## 1. Introduction

**Figure 1:**
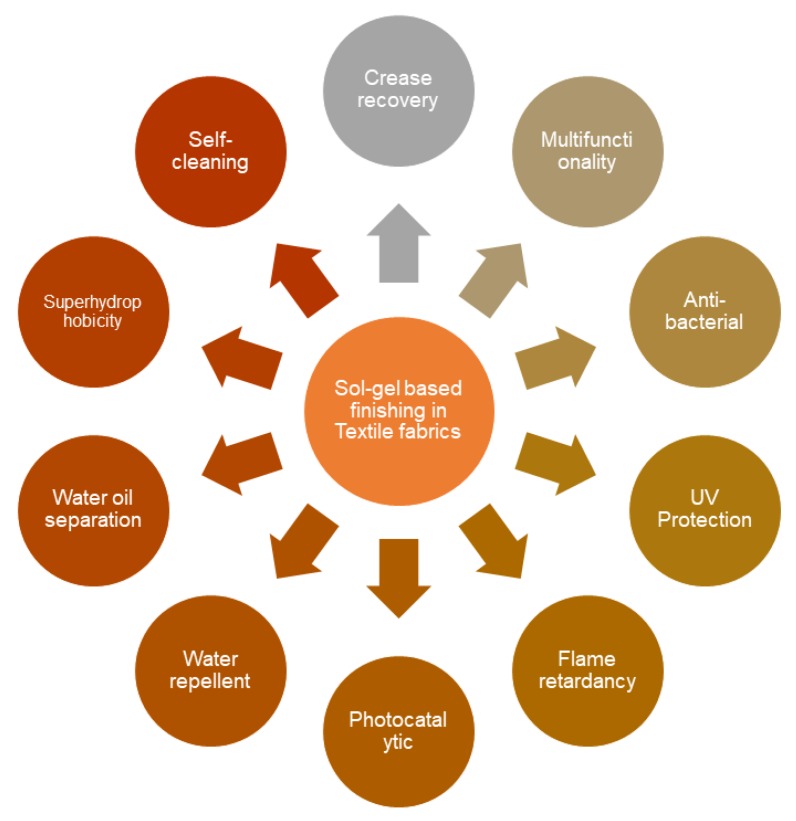
Various applications of sol-gel finishing on textile fabrics.

Textile wet processing consists of three important processes, namely pretreatment, coloration (dyeing or printing) and finishing [1,2]. Pretreatment (i.e. scouring, bleaching) is done first to prepare the textiles for the subsequent process called coloration [3,4]. After coloration, the textiles are subjected to the finishing process according to the end-use or consumer requirements [1,2,5,6]. As mentioned, the finishing process is the last chance to provide added value [7]. The finishing process provides special functional properties to the textiles such as flame-retardancy, water-repellency, water-proof, antimicrobial, soil/stain resistance, etc. [8]. Textile finishing can be classified according to different factors based on durability (durable or semi-durable), chemical (wet processing), mechanical (dry or physical treatments such as brushing, shearing, raising), aesthetic (modify the hand/ drape) and functional finishes. Generally, chemical finishing involves the addition of different chemicals based on the intended textile end-use [7]. Among the different finishing techniques, chemical finishing has been widely studied due to the current trends together with the customer requirements for high-tech or high-performance applications [9,10,11,12]. In the past two decades, sol-gel- assisted textile finishing has played a vital role in the development of novel applications to improve the basic properties of textiles. Generally, sol-gel-based textile finishing has more advantages to overcome the shortcomings of conventional finishing techniques [13,14,15,16,17]. The main advantages are eco-friendliness, less chemical utilization, low-temperature treatment, low toxicity to human health, protection of the inherent properties of textile materials, and the possibility to adjust the thickness of the coating and long-lasting properties of finished fabrics. Some types of sol-gel systems also have bacteriostatic or antibacterial effects [16,17,18,19,20,21,22]. These systems are anatase-modified photoactive TiO_2_ coatings and sol-gel coatings with colloidal metals or metal compounds embedded in them, such as silver, silver salt, copper compound, zinc or quaternary ammonium salt [18], so sol-gel technology can be applied to textiles to develop various functional finishes with antibacterial [23,24,25,26,27,28,29,30,31,32], water repellent [33,34,35,36,37,38], superhydrophobic [39,40,41,42,43,44], oil/water separations [45,46,47,48,49,50,51,52,53], flame-retardant [54,55,56,57,58,59,60,61,62] multi-functional [63,64,65,66,67,68], ultraviolet (UV) protection [69,70], self-cleaning as well as soil-repellency [18,71,72], photocatalytic [73,74], wear & abrasion resistance properties [75]. The main aim of this review paper is to describe the history, synthesis, application and progress of sol-gel finishing in the textile industry. The various applications of sol-gel techniques in textile finishing are summarized in Figure 1.

### 1.1. Sol-Gel Technology: History, Chemistry and Synthesis

Sol-gel technology has tremendous potential due to the combination of novel materials with a high degree of homogeneity at the molecular level with excellent physical and chemical properties [76,77]. In ancient times in China, tofu was prepared by utilizing this technology and hence it is not a new technology [78,79]. In the mid-19th century, sol-gel technology was used for preparing one-component compounds using sols and gels [80,81]. In the process of making glass, the sol-gel process requires less temperature compared to the conventional high-temperature melting method [82,83]. The method of forming a gel by mixing SiCl_4_ and ethanol and hydrolysis in humid air was discovered by Ebelmen in 1846 [84,85]. The sol-gel method was successfully used to produce SiO_2_-B_2_O_3_-A1_2_O_3_-Na_2_O-K_2_O multi-component glasses which were analyzed by Dislich [86], thus creating great curiosity towards sol-gel technology. In 1981, the first international workshop on glasses [77,79,81,87] and ceramics from gels was held, which helped to further develop sol-gel technology. Sol-gel technology is being used widely since 1980 in the process of synthesizing superconducting materials [88], functional ceramic materials [89], nonlinear optical materials [90], catalysts and enzyme carriers [91,92], porous glass materials and other materials [93,94]. It was a milestone in the history of materials science in which many papers and patents broadly utilizing surface coating and other aspects were published [95,96]. The solution, sol or gel, solidifies the compounds of metal-organic or inorganic (precursor) to form a sol or gel state, followed by the development of an oxide by heat treatment. The polycondensation reaction transforms Si-OR- and Si-OH-comprising species into siloxane compounds which is the basic chemical principle of sol-gel treatment of silica-based materials. Corner sharing connects this to SiO_4_ tetrahedra (or RSiO_3_ tetrahedra in hybrid materials) from a structural point of view. In order to achieve a stable gel, it is essential to maximize the number of siloxane bonds (-Si-O-Si-) and subsequently minimize the number of silanol (Si-OH) and alkoxo (Si-OR) groups [18,97,98,99].

#### Synthesis of Sol-Gels

**Figure 2:**
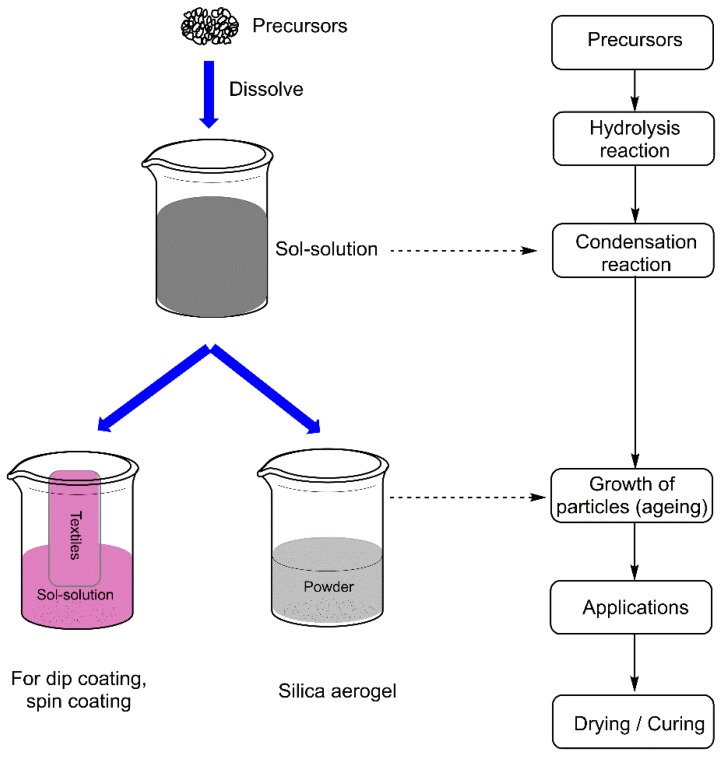
Steps involved in a sol-gel process.

Sol-gel is a wet treatment process, and for sol-gel synthesis, a homogeneous solution is formed by dissolving the precursor in a solvent reaction (water or an organic solvent) which is the foremost step, irrespective of whether the raw material is either an inorganic salt or metal alkoxide [82,97]. Sol-gel processes can be divided into five stages called hydrolysis, condensation (gelation), gel aging, applications and curing. A schematic representation of the sol-gel process is presented in Figure 2.

The sol is formed by hydrolyzing or alcoholizing the solute or solvent whereby the resultant product is divided into little nanometer-sized particles [100,101,102,103]. The basic reactions of sol-gel formation are given below [101]:

- *Solvation:* the ionizable precursor-solvent unit $M{\left(H_{2}O\right)}_{n}^{z+}$ (where z is the valence of the M ions) is formed by the metal cation M^z+^ of the metal salt attracting the molecules of water and further H^+^ is released due to its strong tendency to maintain its coordination number:
  (1) $${M(H}_{2}{O)}_{n}^{z+}\to {M(H}_{2}{O)}_{\left(n-1\right)}{\left(\mathrm{OH}\right)}^{{\left(z-1\right)}^{+}}{+H}^{+}$$
- *Hydrolysis*: A non-ionized molecular precursor reacts with water, such as a metal alkoxide M(OR)_n_ (n is the valence of the metal M, and R represents an alkyl group).
  (2) $${M(\mathrm{OR})}_{n}{+\mathrm{xH}}_{2}O\to {M(\mathrm{OH})}_{x}{\left(\mathrm{OR}\right)}_{n-x}+\mathrm{xROH}$$
- *Polycondensation*:
  (3) $$\text{-}M\text{-}\mathrm{OH}+\mathrm{OH}\text{-}M\to {\text{-}M\text{-}O\text{-}M+H}_{2}O$$
  (4) -M-OH+RO-M→-M-O-M+ROH
  (5) $${M(\mathrm{OR})}_{n}+\mathrm{mXOH}\to {\left[M(\mathrm{OR}\right)}_{n-m}{\left(\mathrm{OX}\right)}_{m}]+\mathrm{mROH}$$

### 1.2. Factors Affecting the Sol-Gel Reaction

#### 1.2.1. Precursor Properties

Metal ion radius, electronegativity, coordination number, etc., all affect the nature of the metal alkoxide in the solvent. The hydrolysis and polycondensation reaction are determined by the number of partial positive charges of the metal atom and the ability of the metal atom to increase its coordination number. The coordination number increases strongly as the positive charge of a metal atom increases, and the reaction rate is faster [104].

#### 1.2.2. Temperature and Time

The rate of hydrolysis of alkoxides accelerates as the reaction temperature increases. To reduce the reaction time alkoxides (such as silicon alkoxide) are often manipulated under heating due to their low hydrolysis activity [105].

#### 1.2.3. Solvent

The activity of alkoxide hydrolysis is changed due to the exchange of the -OR groups in the alkoxide with -OR’ groups in the alcohol solvent. Hence, depending on the selected solvent, the rate of hydrolysis and gelation time of the same alkoxide may vary. Drying of wet gels is also affected by the solvent type. The rate of drying is higher when the saturated vapor pressure of the solvent is high but this may cause cracking of the gel [19,106].

#### 1.2.4. Properties and Concentrations of Catalyst

Even though the silicon alkoxide is hydrolyzed in the absence of the catalyst, the use of catalysts can increase the rate of hydrolysis and the hydrolysis is more thorough. The most commonly used catalysts are hydrochloric acid and ammonia [107,108].

#### 1.2.5. Ratio of Water to Metal Alkoxide

The ratio of the amount of water to precursor is measured by the water amount and is represented usually by the symbol *R*. It directly influences the structure of the alkoxide-hydrolyzed polycondensation product [109]. The molecules of alkoxide are less hydrolyzed alkoxys due to the less amount of water which is the formation of hydroxyls by less hydrolysis, and a low degree of crosslinking is produced by the polycondensation between the partially hydrolyzed alkoxide molecules. On the contrary, a highly cross-linked product is easily formed with more amount of water. The quantity of water is also meticulously connected to the viscosity and gelation time of sol. An increase in *R* leads to an increase in the viscosity of the sol and the shortening of gelation time due to the fact the amount water is less than the stoichiometric amount of water (*N*) required for hydrolysis. Since the water amount maximizes the degree of crosslinking, the degree of polymerization of the polycondensate also increases. However, when the water quantity exceeds *N*, with increasing *R* the viscosity will be reduced and the gelation time will be prolonged due to the excess amount of water reducing the concentration of polycondensate. Additionally, an excess amount of water also affects the subsequent drying process in causing shrinkage and drying stress of gels and prolonging the drying time [108].

## 2. Progress in Sol-Gel Finishing on Fabrics

### 2.1. Water Repellent

**Figure 3:**
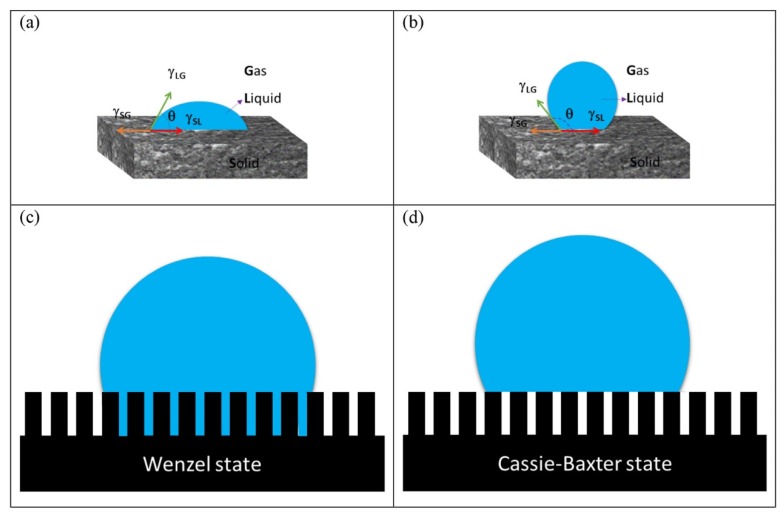
(**a**) Droplet on the flat surface with a contact angle lower than 90°; (**b**) higher than 90°; (**c**) Wenzel model; (**d**) Cassie-Baxter model (Reproduced from [113], with kind permission of Elsevier).

In the conventional method, a fluorocarbon compound can be used for water repellent finishing. However, due to the harmful effects of fluorocarbon components on the environment as well as wearers, many researchers have studied non-fluorinated sol-gel finishing as an alternative. Based on the Wenzel (Figure 3a) [110] and Cassie-Baxter (Figure 3b) [111] theories, superhydrophobic textiles can be created by sol-gel finishing through generating low surface energy and a rough surface on the fibers. Generally, water contact angle (WCA) measurements are used to specify the wettability and performance of water-repellent fabrics [112]. Wettability defines how the liquid can spread out on the solid or liquid substrate. Tiny liquid droplets of known surface tension are applied on the substrate surface and then a measurement of the contact angle is taken (Equation (6) and Figure 3). For water, the surface is defined as hydrophilic when WCA < 90° and hydrophobic when WCA > 90°.

(6) $$\gamma_{SG}=\gamma_{SL}+\gamma_{LG}\cdot c\mathrm{os}\theta$$

(7) $$\gamma_{LG}=\gamma_{LG}^{d}+\gamma_{LG}^{p}$$

(8) $$\gamma_{SG}=\gamma_{SG}^{d}+\gamma_{SG}^{p}$$

(9) $$\gamma_{LG}\cdot \left(1+c\mathrm{os}\theta \right)=2\sqrt{\gamma_{SG}^{d}\cdot \gamma_{SG}^{d}}+2\sqrt{\gamma_{SG}^{p}\cdot \gamma_{SG}^{p}}$$

**Figure 4:**
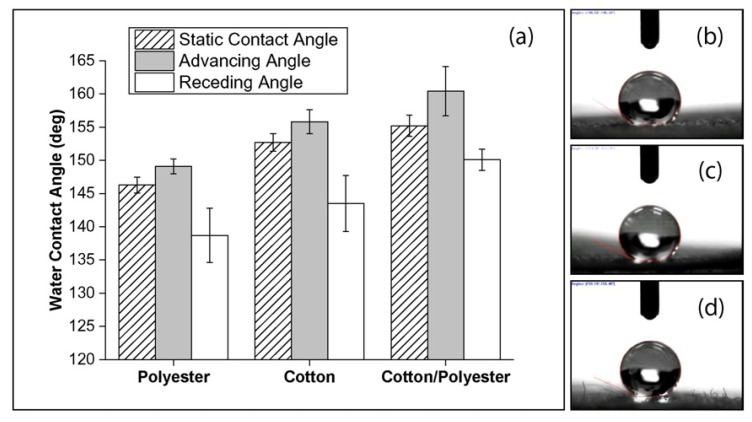
(**a**) Water contact angle of finished fabric; (**b**) droplet on polyester; (**c**) cotton; (**d**) blend of polyester cotton fabric (Reproduced from [40], with kind permission of Elsevier).

**Figure 5:**
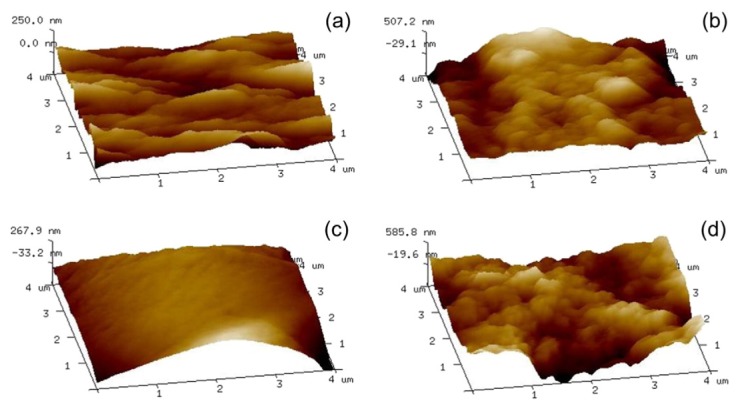
AFM images of pristine (**a**) cotton; (**b**) coated cotton; (**c**) pristine cotton/polyester and (**d**) coated cotton/polyester (Reproduced from [40], with kind permission of Elsevier).

Zhao et al. [40] used a nano-sol containing SiO_2_ nanoparticles, 3-aminopropyltriethoxy silane (APTES) and hexadecyltrimethoxysilane (HDTMS) and sprayed the nano-sol on cotton, cotton/ polyester and polyester fabrics until they were wet and later the coated fabrics were dried at room temperature for one night. The treated fabrics showed superhydrophobicity with WCAs larger than 150° (Figure 4). The surface roughness of treated fabric measured by atomic force microscopy (AFM) is shown in Figure 5. Increasing the concentration of HDTMS or silica nanoparticles results in a higher CA and lower surface energy than untreated fabric. Cotton fabrics and polyester fabrics did not lose their superhydrophobic properties after five washing and drying cycles. In addition, the fabric remained hydrophobic after 600 abrasion cycles.

**Figure 6:**
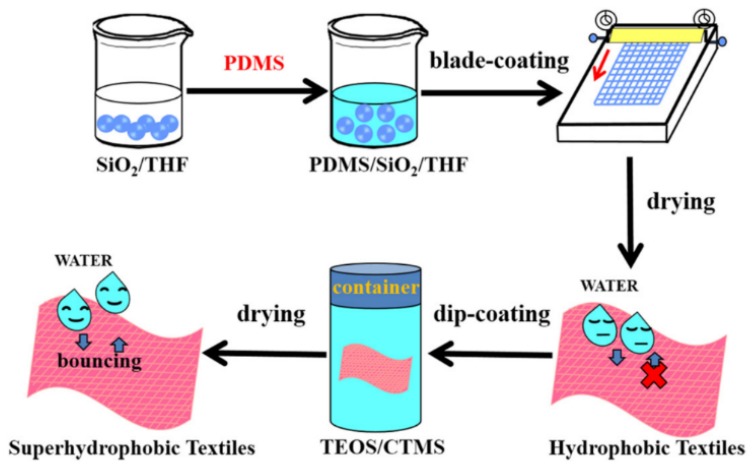
Illustration of the fabrication of superhydrophobic textiles with water pressure resistance (Reproduced from [42], with kind permission of Elsevier).

**Figure 7:**
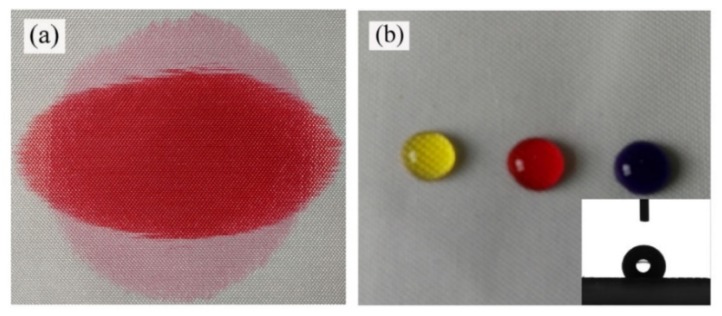
(**a**) Colored water droplet on pristine PET fabric; (**b**) PDMS/SiO_2_-PET fabric after four times blade coating (Reproduced from [42], with kind permission of Elsevier).

Xue et al. [42] coated polyester fabric with polydimethylsiloxane (PDMS) and then worked it up by blade coating with an alcohol solution containing tetraethyl orthosilicate (TEOS) as well as cetyltrimethoxysilane (CTMS). The schematic process is shown in Figure 6. The hydrophobicity of the fabric coated with PDMS-silica nanoparticles showed WCAs above 90° and the superhydrophobicity of TEOS and HDTMS post-treated fabrics provided a WCA of 162.5° and sliding angle (SA) of 5.4° (Figure 7). The treated fabric was resistant to hydrostatic pressures up to 38.6 kPa, and as the number of coatings increased, the sliding angle increased slightly. Their contact angle and water resistance did not change significantly after alkali and acid treatment or UV irradiation at different pH values. However, the bonding force between the coating and the fabric was insufficient and the water pressure resistance of the fabric was reduced to 10 kPa after a 10-cycle abrasion test.

**Figure 8:**
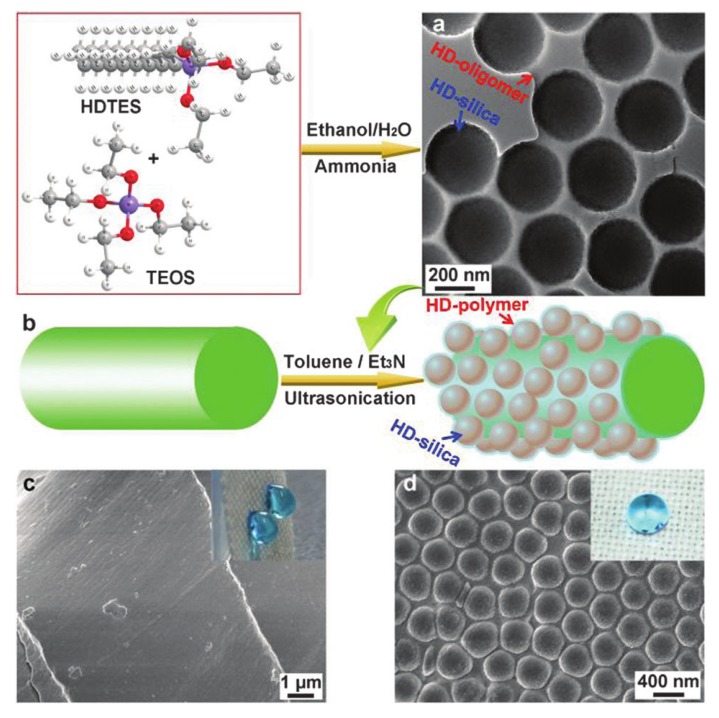
(**a**) Preparation of HD-oligomer/HD-silica nanocomposite; (**b**) Scheme for the super-hydrophobic fabric preparations; (**c**) SEM images of pristine and (**d**) HD-polymer/HD-silica coated superhydrophobic wool fabrics (Reproduced from [41], with kind permission of Royal Society of Chemistry).

Zhang et al. [41] prepared a durable and robust superhydrophobic wool fabric by using fluoro-compound free organosilanes. The schematic process is shown in Figure 8. The hydrophobic wool fabric has been modified into superhydrophobic (Figure 8c) by the usage of nanocomposite composed of an oligomer of hexadecyltriethoxylsilane (HDTES) (HD-oligomer) and HDTES modified silica nanoparticles (HD-silica) that was prepared in advance via a modified Stöber method. HD-silica/HD-oligomer nanocomposites were applied on the wool fabric by a simple dip-coating method achieved using toluene as solvent and triethylamine as a catalyst, and ultrasonic treatment was carried out to prepare a wool fabric having excellent abrasion resistance, environmental stability and superhydrophobicity. The prepared samples were proved to have excellent mechanical resistance after being scratched by a scalpel. After 10 washes, the superhydrophobic property of the finished fabrics remained good.

Przybylak et al. [114] used a bifunctional fluorosilsesquioxane with different functional groups to modify the surface of cotton fabric and it showed superhydrophobic properties with a contact angle of 151° and resistance to 10 washing cycles. Xue et al. [44] prepared superhydrophobic cotton fabrics with TiO_2_ particles through a sol-gel coating process. The incorporation of TiO_2_ particles enhances not only the superhydrophobicity, and also the UV shielding properties. This method has an additional advantage in that it does not require expensive equipment to carry out the process, as compared to other conventional processes.

Onar and Mete [115] studied a two-step method to prepare water repellent finishes on cotton fabric with ZnO, Al_2_O_3_, TiO_2_ and ZrO_2_ nanoparticles. The cotton fabric surface was first roughened with ZnO, Al_2_O_3_, TiO_2_ and ZrO_2_ nanoparticles, and then modified hydrophobically with HDMS. The results show that the cotton fabric treated with Al_2_O_3_ nanoparticles and HDMS has superhydrophobic properties, and after washing, the fabric can maintain its hydrophobicity. Colleoni et al. [116] treated cotton and polyester fabrics with tetraethoxysilane (OTES) and melamine-based crosslinkers (N,N,N′,N′,N″,N″-hexakis(methoxymethyl)-1,3,5-triazine-2,4,6-triamine (MF)). Cotton fabrics and polyester fabrics were found to have good hydrophobicity with contact angles of 130° and 150°, respectively. Teli et al. [43] synthesized SiO_2_ nanoparticles by a simple sol-gel technique and they were applied to nylon knitwear by double-dip and double nip coating with a wet pickup of 80%. After the impregnation, the treated fabric was dried and cured. The modified nylon knit fabric had superhydrophobic properties with a WCA of 151°. The fabric also exhibited excellent UV protection with an ultraviolet protection factor (UPF) of 279.68 (i.e., UPF 50+). After 10 wash cycles, it still had hydrophobic and UV protection properties. De et al. [117] applied nanogels composed of 3-glycidoxypropyltrimethoxysilane (GPTMS) and a fluorine-containing alkyl functional water-based oligosiloxane (FAS, Dynasylan F8815) on silk fabrics by a sol-gel method using an acid catalyst. After finishing, the fabric was tested for 8500 abrasion cycles. The results confirm the oleophobicity (close to 123° for the 4th oil contact angle) and high hydrophobicity (WCA 148°). After analyzing the oil resistance and the water repellency of fabric, it was confirmed that the fabric was still hydrophobic (CA of 130°) and oleophobicity (CA of 100°) after 15 washing cycles. Vasiljevic et al. [118] prepared highly oleophobic and superhydrophobic cotton fabrics by a three-step sol-gel method. The first step was to prepare a cotton fabric treated with SiO_2_ nanoparticles which have an average particle diameter (50 ± 15 nm, 230 ± 20 nm and 780 ± 30 nm) by the Stöber method based on a sol-gel process; the second step is to grow the silicone layer in situ in the alkaline TEOS solution.; the third step was filling with a fluorine-containing alkyl functional water-based oligosiloxane (FAS, Dynasylan F8815). The fabric presented a low slip angle (2°), high oleophobicity (grade 6 oil, contact angle close to 150°) and superhydrophobicity over 10 washes. Zhou’s group [119] fabricated a durable superhydrophobic fabric coating via dip-coating fluoroalkyl silane-modified SiO_2_ nanoparticles, PDMS and a fluoroalkyl silane mixture. The coated fabric exhibited excellent durability against strong acid, strong alkali, and the coating still showed great superhydrophobicity with WCA above 160° after 500 washing cycles and severe abrasion cycles, respectively. Cao et al. [120] prepared the fluorine-free superhydrophobic fabric by simple dip-coating method. The PDMS coated fabric showed a WCA of 160° and in addition it provided the self-cleaning properties and oil/water separation abilities.

### 2.2. Oil and Water Separations

**Figure 9:**
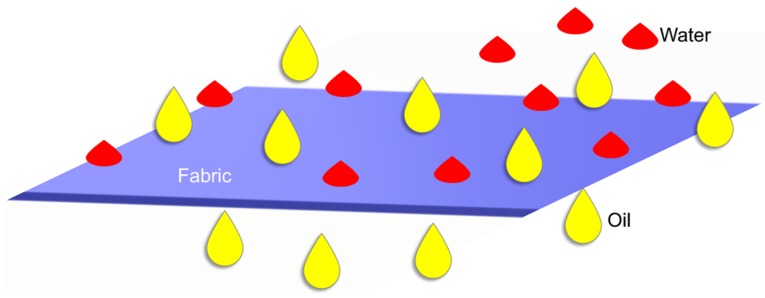
Oil/water separation principle.

Crude oil products have been one of the necessities in our life. However, while mining and transporting oil spillages [121] can occur which can cause great losses to the economy and pollution of the environment [122]. Water pollution created by this threatens the daily life of organisms [45,121,122,123,124,125]. As an example, the oil spillage in the Gulf of Mexico in 2010 of nearly 720 thousand tons of crude oil affected the economy terribly and harmed all kinds of creatures living in the sea [126]. Usually mining operations discharge around 140 m^3^ of oil into waters [123]. Hence, there is a great necessity to provide an effective and speedy way to purify polluted water as its scarcity is now a crucial problem of our world. Cotton fabrics [127], non-woven fabrics [128], wood cellulose nanofibers [129], melamine sponges [53], aerogels [46] and activated carbon fibres [130] are common oil-absorbing materials which can be effectively utilized in cleaning and collecting crude oil. However, due to their high hydrophilicity, poor oil/water selectivity, low oil/water separation efficiency and difficulty of reuse [131], these measures cannot provide a satisfactory solution. Subsequently there is a high demand for oil-absorbing materials with low cost, efficient selectivity, recyclability and high oil absorption rates. Researchers have developed materials with special wettability to separate oil/water mixtures, subject to the different wettability of water and oil, such as superhydrophobic and superoleophilic materials [132], superhydrophilic and underwater superoleophobic materials [133], superhydrophilic and superoleophobic materials [49] which offer solutions to the problem. In general, “oil removing” is one of the strategies to pass through and collect oil when a hydrophobic and oleophilic surface repels water [47,48]; and the other strategy is “water-removing” where hydrophilic and oleophobic materials permit water to pierce a surface while preventing the oil phase from passing entirely (Figure 9) [51].

**Figure 10:**
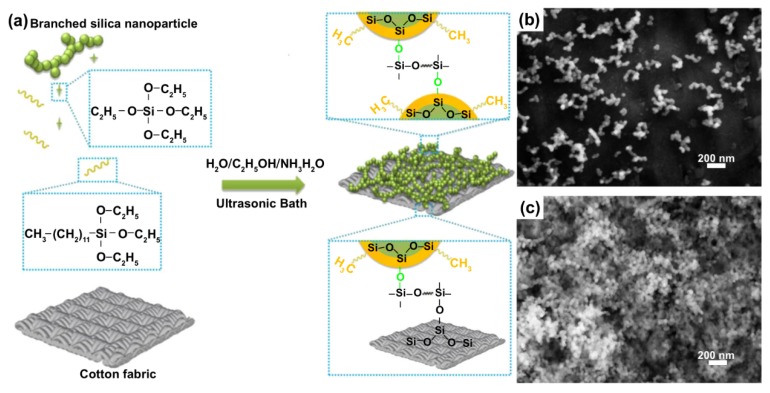
(**a**) One-pot fabrication process of superhydrophobic cotton fabric; (**b**) branched silica nanoparticles and (**c**) porous nanostructure of branched silica nanoparticles (Reproduced from [52], with kind permission of Springer).

**Figure 11:**
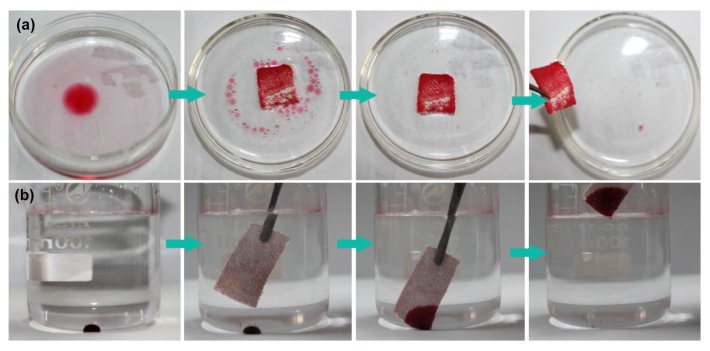
(**a**) Performance of removing oil floating on water and (**b**) heavy oil underwater (Reproduced from [52], with kind permission of Springer).

Shang et al. [52] developed a superhydrophobic and superoleophilic cotton fabric with the help of sono-chemical irradiation as well as silanes containing silica nanoparticles. The finished cotton fabric showed a CA of 159 ± 1.2°. The oil separation efficiency from water could reach a maximum at 98.2%, that was reduced to 94% after 30 separation cycles. The schematic representation of superhydrophobic and superoleophilic fabric is shown in Figure 10. A branched silica nanoparticle was used to develop the nano-porous structures on the fabric, as shown in Figure 10b,c. The oil removal ability of the treated cotton fabric was good and the result is shown in Figure 11. As well as oil floating on water, the superhydrophobic cotton fabric could also absorb chloroform underwater. The treated fabric absorbed chloroform immediately when the fabric was placed in contact with chloroform.

**Figure 12:**
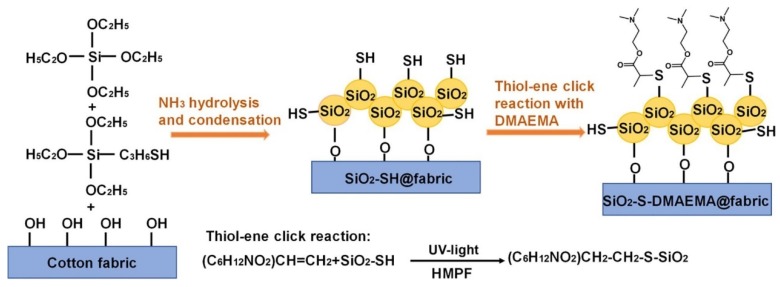
Schematic illustration of the formation of SiO_2_-S-DMAEMA on cotton fabric (Reproduced from [135], with kind permission of Elsevier).

**Figure 13:**
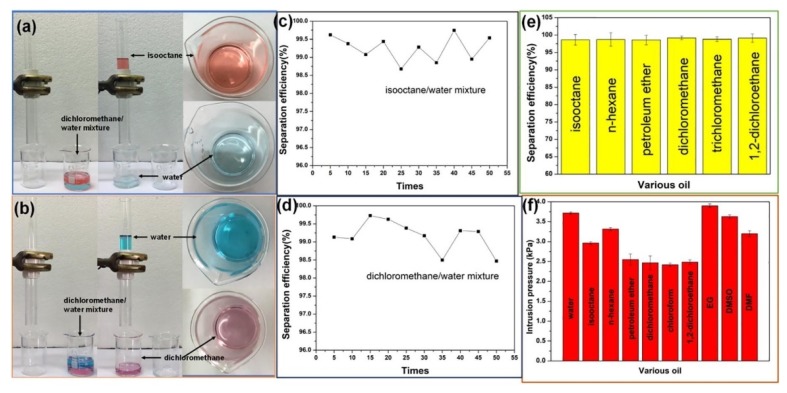
(**a**) Performance of treated fabrics on light oil/water separation; (**b**) heavy oil/water separation; (**c**) 50 times recyclable separation efficiency of isooctane/water mixture; (**d**) 50 times recyclable separation efficiency of dichloromethane/water mixture; (**e**) separation efficiency of the fabric for various oil/water mixtures and (**f**) intrusion pressure of the fabric (Reproduced from [135], with kind permission of Elsevier).

A superhydrophobic cellulose membrane for efficient oil/water separation was prepared by Xie et al. [134]. A one-step facile sol-gel strategy was applied to obtain micro/nano hierarchical structures and low-surface-energy chemical modification from TEOS and hexadecyltrimethoxysilane (HDTMS). From X-ray photoelectron spectroscopy (XPS) analysis, the authors detected Si groups after TEOS and HDTMS coating. WCA was increased with increasing dosage of HDTMS. The treated membrane had superhydrophobicity and superoleophilicity which confirmed its oil/water separation ability. Chen et al. [135] fabricated a superhydrophobic and superoleophobic fabric by a two-step approach involving a sol-gel process followed by grafting. The sol-gel process can help to deposit SiO_2_-SH particles on the fabric surface and is followed by grafting 2-(dimethylamino)ethyl methacrylate (DMAEMA) on the fabric. A detailed schematic illustration of this process is shown in Figure 12. The treated fabric shows better oil/water separation, water-in-oil and oil-in-water emulsion separation properties, even after various harsh treatments (Figure 13).

**Figure 14:**
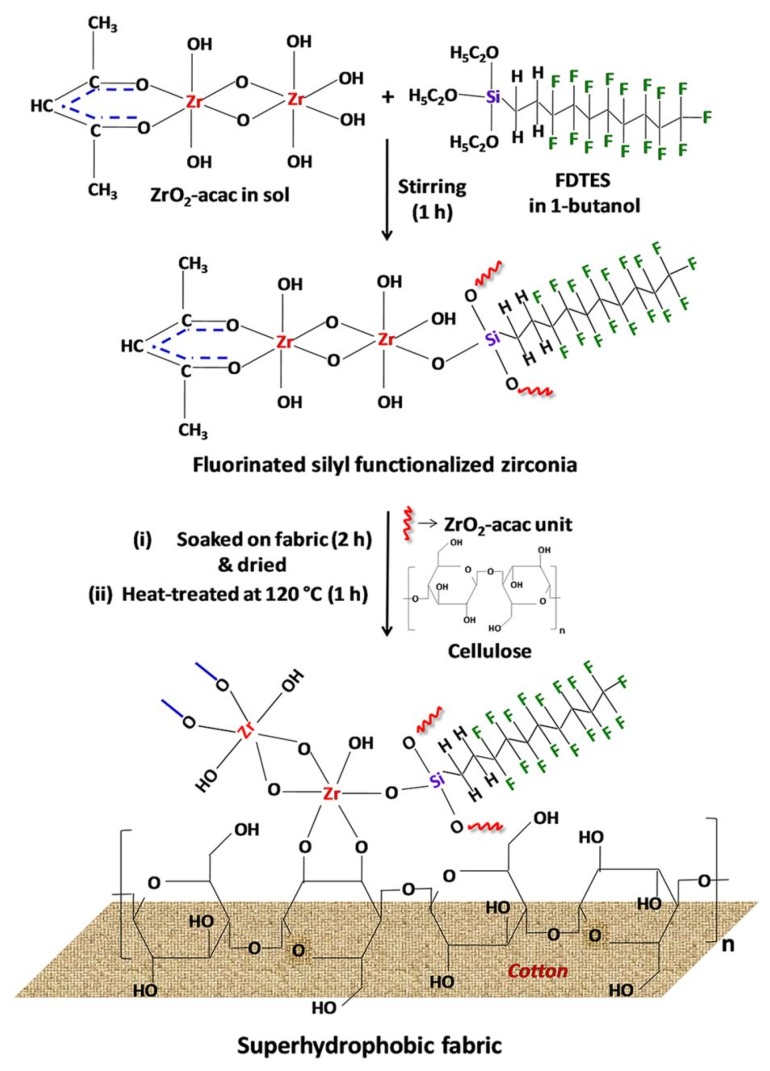
Schematic representation of fluorinated silyl functionalized zirconia sol-gel coated fabric (Reproduced from [137], under a Creative Commons Attribution 4.0 International License).

**Figure 15:**
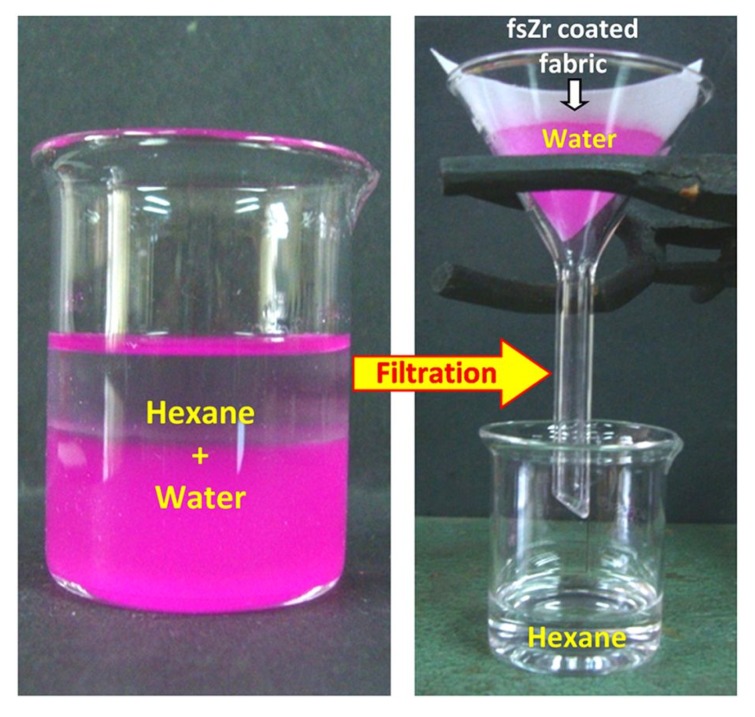
Hexane-water separations (Reproduced from [137], under a Creative Commons Attribution 4.0 International License).

Xu et al [136] produced a superhydrophobic and superoleophilic glass-fiber fabric by a simple sol-gel process using triethoxymethylsilane (MTES) as precursor. The prepared fabric can effectively separate micron-sized surfactant-stabilized water-in-oil emulsions solely driven by gravity. Most importantly, this fabric can withstand acid/alkaline and high-temperature environments. Besides, the separation efficiency of as-prepared glass-fiber fabric remains high even after being used 20 times. Hence, this simple, efficient, and low-cost preparation of fabric with special wettability shows potential in industrial applications. Das et al. [137] developed a zirconia-based superhydrophobic and superoleophilic coating on cotton fabric (Figure 14). The resultant fabrics showed excellent water repelling and superoleophilic properties with chemical stability. In this case the authors performed the water separation experiments on coated “filter cloth” as shown in Figure 15. When a vigorously stirred mixture of hexane-water was poured onto the treated cotton fabric, hexane immediately spread and freely permeated through the fabric at atmospheric pressure and, rapidly accumulated into the bottom of the beaker. It can be clearly seen in Figure 15 that there was no water in the separated hexane. On the other hand, the water (red colored by Rhodamine B for visibility) still remained on the textile surface.

**Figure 16:**
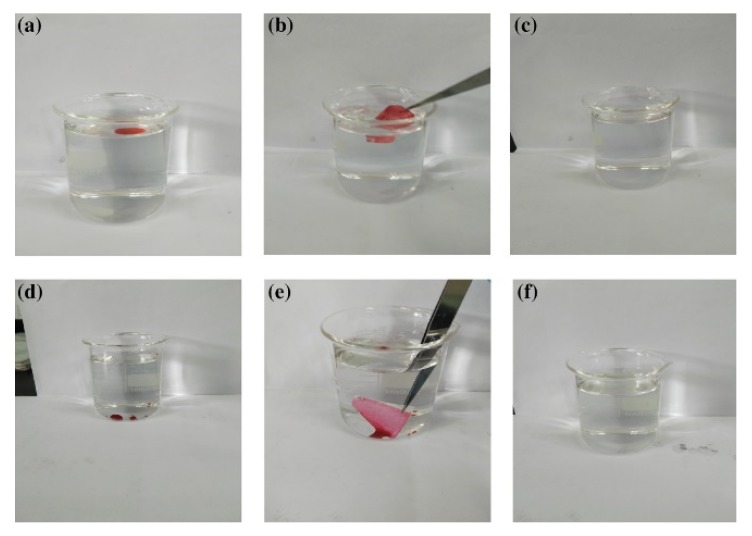
(**a**–**c**) Fluorinated polymeric sol-gel incorporated fabric on hexane removal and (**d**–**f**) chloroform with the help of water (Reproduced from [138], with kind permission of Wiley).

Superhydrophobic and superoleophilic cotton fabrics were successfully prepared with fluorinated polymeric sol-gel precursor (PHFBMA-MTS) by Jiang et al. [138]. The coated fabrics showed superhydrophobic properties with a high-water contact angle of 154.1° and superoleophilic properties with an oil contact angle of 0°. Moreover, the coated fabrics maintained their superhydrophobicity even after ultrasonic treatment, as well as exposure to organic solutions and acidic solutions. Thus, the coated fabrics were successfully applied to separate oil/water mixtures with separation efficiencies of up to 99.8%. More importantly, the separation efficiency experienced no significant change after 20 oil/water separation cycles. The sorption capability of finished cotton fabric is demonstrated with hexane and chloroform in Figure 16a–c,d–f, respectively. 

**Figure 17:**
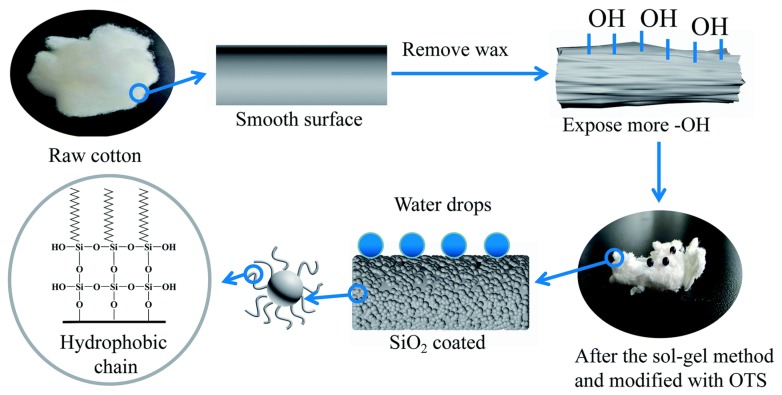
Schematic illustration of the modification cotton fiber (Reproduced from [139], under Creative Commons Attribution 3.0 International License).

Lv et al [139] developed a superhydrophobic/superoleophilic cotton fabric specially as an oil absorbent with the help of TEOS. Figure 17 shows a schematic illustration of the fabrication process of SiO_2_ particles on the surface of the modified cotton fiber via the sol-gel method. 

Because of the coverage of a small amount of plant wax on the surface of the raw cotton fiber, it is difficult for it to absorb more oil. The primary function of the NaOH pretreatment is to remove this plant wax and to improve the interface bonding properties. After coating the SiO_2_ particles, a rough fiber surface structure was created. The sorption capacities of the raw cotton and the cotton that was modified with OTES in pure water and in an oil system were measured by using various liquids (artificial seawater, crude oil, diesel oil, peanut oil, and lubricating oil). The adsorption capacity of treated fabric is expressed as follows (according to ASTM F-726-12):

(10) $$S_{S}=\frac{S_{ST}-S_{O}}{S_{O}}$$

where *S_S_* is the sorption degree (g (liquid)/g (sorbent)), and *S_O_* and *S_ST_* are the mass of the cotton fiber before and after sorption, respectively.

### 2.3. Flame Retardancy

**Figure 18:**
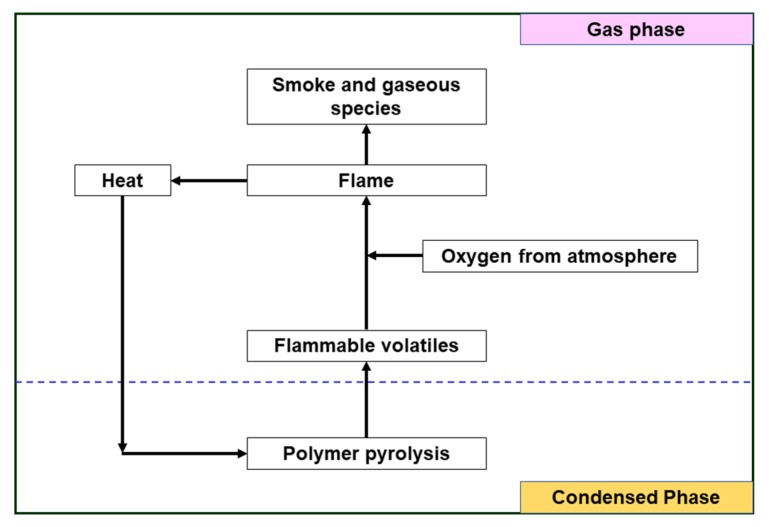
Potential modes of flame-retardant action in a self-sustaining polymer combustion cycle (Adapted from [145] under Creative Commons Attribution 4.0 International License).

Generally speaking, flaming combustion is a gas-phase oxidative process: for this reason, it needs oxygen (or air) from the atmosphere. Therefore, before undergoing flaming combustion, a polymer has to degrade; the degradation promotes the formation of combustible volatile species that can mix together with atmospheric oxygen and fuel a flame. Because of the highly exothermic character of the flame, when the heat transferred to the material surface is enough, it may give rise to further degradation, hence promoting a self-sustaining combustion cycle (Figure 18). The reduction of flammability of textiles [54,55,56,140,141,142,143,144] by the sol-gel process was widely explored in the past decade. Organic and inorganic compounds were effectively trapped with varied functionalities on diverse substrate surfaces. This offers prospects for functional finishing of textiles substrates with diminished fading under normal handling conditions.

The sol-gel method can significantly reduce the high chemical concentration of materials used in conventional methods (close to 300–500 g/L), or use halogen-free chemicals, so an ecologically and economical way to produce flame-retardant finished fabrics can be obtained. Grancaric et al. [146] improved the durability of flame-retardant finishes on cotton fabrics by using diethylphosphoryl-ethyltriethyloxysilane (DPTES) as a sol-gel precursor and monoethanolamine as a neutralizing agent for the acidic conditions of the DPTES with/without addition of OTES and 3-glycidoxypropyl-triethoxysilane (GPTES). The results showed that the finished sample was in a self-extinguishing state with a limiting oxygen index (LOI) of 29%. The residual amount of treated fabric was observed to increase from 37.5% to 60% compared to untreated fabric at the maximum thermal decomposition temperature, and the total heat of the treated fabric was reduced from 124.2 kJ/g to 4.2 kJ/g. The char formation caused by the thermal shielding effect of the silica phase and the synergistic effect of phosphorus and nitrogen is the cause of the flame retardancy and thermal behavior of the cotton fabric. However, the LOI value of the fabric sample after one wash was reduced from 29% to 21%. Boukhriss et al. [147] used a sol containing chloropropyltriethoxysilane (CPTS), 1-methyl-imidazolium chloride propyltriethoxysilane (MCPTS) and 1-pyridinium chloride propyl triethoxysilane salt (PCPTS) for flame retardant and water-repellent finishing on cotton fabrics. Results showed that the sol-gel finishing improves the flame retardancy and water repellency on cotton.

**Figure 19:**
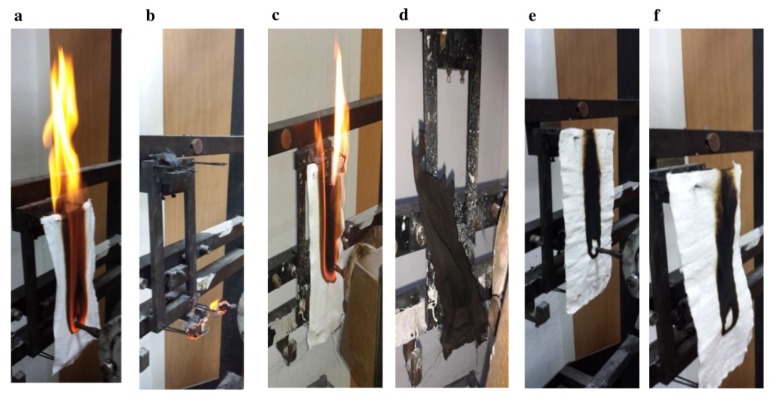
(**a**) Flammability test of untreated cotton fabric after 20 s; (**b**) after 45 s; (**c**) CPTS treated cotton after 20 s; (**d**) after 67 s; (**e**) MCPTS treated cotton after 20 s and (**f**) PCPTS treated cotton after 20 s (Reproduced from [147], with kind permission of Springer).

**Figure 20:**
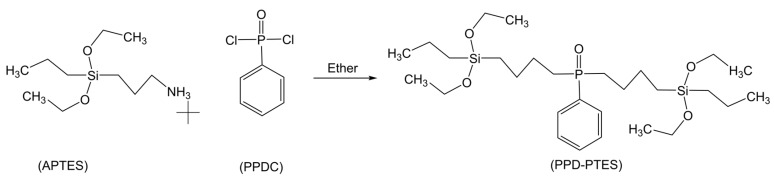
Synthetic route of cotton fabric with hybrid silica for flame retardant finishing (Reproduced from [151], with kind permission of Elsevier).

**Figure 21:**
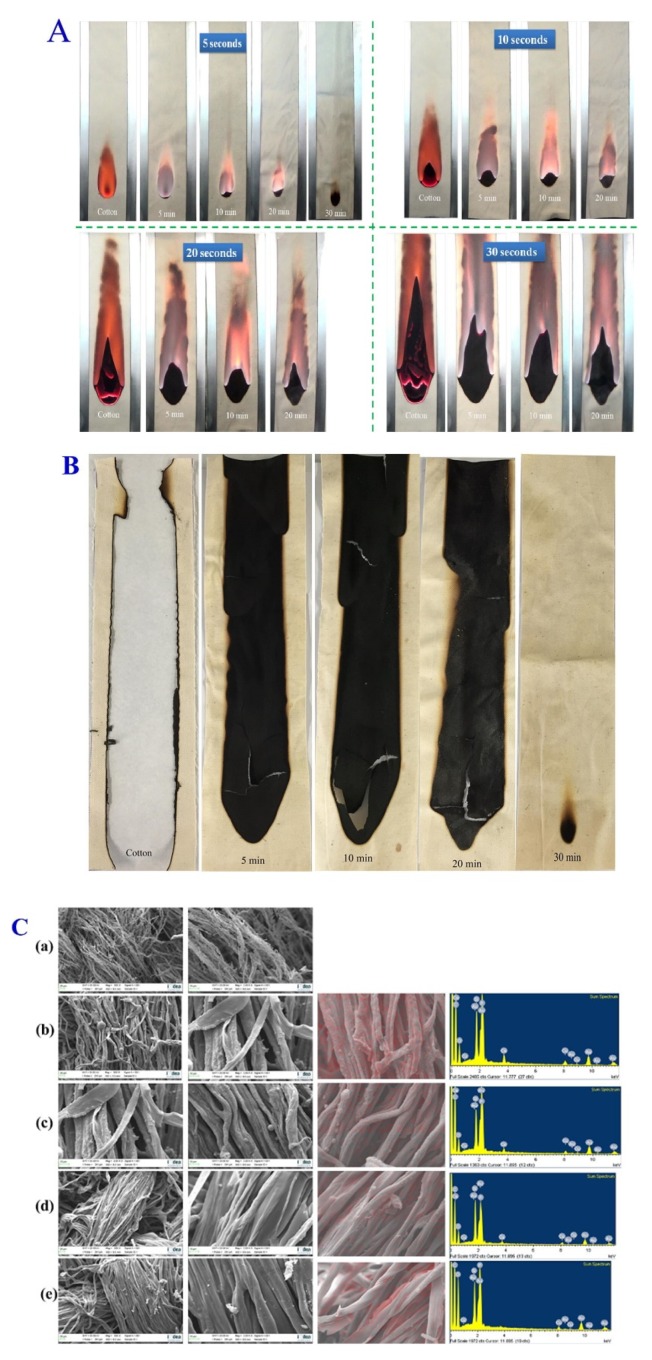
(**A**) Flammability test on untreated and treated cotton fabrics; (**B**) actual images of fabrics after flammability test; (**C**) SEM images and EDX spectra of cotton (**a**), cotton-5 min (**b**), cotton-10 min (**c**), cotton-20 min (**d**) and cotton-30 min (**e**) at different magnification (×500, ×2000, respectively) after burning (Reproduced from [151], with kind permission of Elsevier).

Recent research shows that ionic liquids together with a silica precursor confer good resistance to a direct flame. Figure 19b,d shows the burning of untreated and treated cotton with CPTS for 45 and 67 s, even after removing the flame, respectively. For untreated cotton, the final residue after the test was 1% (in weight) and the one treated with CPTS was 15%. In either way, fabrics made from cotton with [MCPTS]PF_6_ or [PCPTS]PF_6_ do not burn. Figure 19e,f show that treated cotton does not flame during and after the removal of the flame. A maximal final residue of 93% was obtained after the application of a flame for 20 s was recorded. A comparative so these these results suggests that ionic liquids can be considered as extremely good flame-retardants for cotton textile materials. Zhang et al. [148] used TEOS and boric acid to improve the flame retardancy of silk fabrics by a sol-gel process along with 1,2,3,4-butanetetracarboxylic acid (BTCA) as crosslinker to improve the durability of fabric flame retardancy. The results showed that when the intrinsic flame-retardant silk fabric containing P, N or S was treated with BTCA solution and also treated with boric acid containing nanosol as flame retardant the LOI value increased from 25.3% to 32.3%. In addition, the flame retardancy of silk fabrics was resistant to 30 washes. The peak heat release rate (PHRR) of the treated silk fabric was reduced drastically. The total exotherm, mass loss and reduced smoke density were reduced compared to the untreated fabric, which indicates low flammability. In addition, there is no change in the tensile properties of the treated samples, but the hand of the sol-treated samples was significantly reduced. Nylon 6 with thermal oxidative stability was prepared by utilizing 9,10-dihydro-9-oxa-10 phosphaphenanthrene-10-oxide-modified vinyl trialkoxysilane (DOPO-VTS) and tetraethoxysilane by Sehic et al. [149]. Thermal analysis of coated samples reveals that the plating affects the decomposition of PA6 at minimal temperatures, in creating a positive char formation and minimizing overall heat release. Tests conducted through vertical flame spread reveal that carbon carbide fully avoids the dripping of the polymer melt at maximal precursor concentrations in the coating. Deh et al. [150] studied the halogen and formaldehyde free flame retardancy of cotton fabrics treated with phosphoric acid and urea phosphoric acid. They studied the synergistic effect of silicon and nitrogen with TEOS as a precursor. They found that cotton fabrics treated with phosphorylation and sol-gel processes had higher limiting oxygen index values (> 25%., i.e. less than 25 % can be easily flammable) and were resistant to 10 washes. Si, N and P on cotton fabrics help to form charcoal acting as a thermal barrier and reduce the number of by-products that do not promote the dehydration process. Liu et al. [151] developed the organic and inorganic hybrid coating on cotton by using APTES and phenylphosphonic dichloride (PPDC) which produce di-(triethoxysilylpropyl) phenylphosphamide (PPD-PTES) as a flame-retardant material. The synthesis route is shown in Figure 20. PPD-PTES was used to coat the cotton fabric at 5, 10, 20 and 30 min, later it was dried 4 h at 100 °C to stabilize the PPD-PTES mixture on the cotton fabrics. Vertical burning tests were used to measure the flame-retardancy of the treated cotton fabric. The cotton fabric treated with the PPD-PTES blend coating self-extinguishes immediately after removal of the ignition source, while the uncoated cotton fabric is burned off, as shown in Figure 21a. The char area on the fabric treated for 30 min was the smallest (Figure 21b) among the other times. From the scanning electron microscope (SEM) images shown in Figure 21c, it was visible that, untreated fabric contained a loose and cracked residue compared to the treated cotton samples after burning tests. Energy-dispersive X-ray spectroscopy (EDX) analysis confirms the more uniform dispersion of phosphorus than silicone. 

Ren et al. [28] studied the flame retardancy of polyacrylonitrile (PAN) fabrics treated by a one-step treatment with TEOS and polyphosphoric acid (PPA) by simple padding-drying-curing steps. The LOI and char residue increased by 30.1% and 69.48%, respectively. The peak and total heat release (PHRR and THR) of the phosphorus-doped SiO_2_ coated polyacrylonitrile fabric decreased significantly, which was 44.9% and 42.1% for the untreated fabric, respectively. In addition, the peaks in both flue gas production and total smoke production were significantly reduced.

### 2.4. UV Protection and Self-Cleaning

Although ultraviolet rays have some benefits, radiation also can cause sunburn, skin aging, allergies and even skin cancer. Textiles can provide effective protection against damage-causing UV radiation. Studies reveal that even cosmetic textiles can protect the skin at the very least. TiO_2_ and ZnO nanoparticles are imbedded into a textile material by a sol-gel method to achieve UV protection. Protection against UV radiation is high by this design. Most of the outfits worn during the time of highest UV exposure in summer are light and colorless materials. Consequently, when determining how to afford the highest protection the type of textile produced must be noted. UV protection can be obtained by incorporating TiO_2_ and ZnO nanoparticles into a textile material by a sol-gel method. Besides UV protection, the anatase phase of TiO_2_ nanoparticles has photocatalytic activity and can provide additional properties such as self-cleaning and antibacterial functions through photocatalytic reactions. Liu et al. [152] used titanium butoxide (IV) (TBT) and tetraethyl orthosilicate as precursors, and 1,2,3,4-butanetetracarboxylic acid (BTCA) as a crosslinking agent along with citric acid triethanolamine salt and tributyl citrate to modify two wool fabrics (raw wool and Kroy craft wool fabric). The self-cleaning properties of the fabrics were evaluated by a photodecomposition test with methylene blue. The treated wool fabrics showed a better self-cleaning property than Kroy craft wool fabrics and the UPF was 356, which is lower than that of Kroy wool fabric (992). The TiO_2_ nanoparticle-treated cotton fabric produced by Dhineshbabu et al. [153] by a sol-gel method has UV protective, antibacterial and flame retardant properties. They found that the fabric samples had UPF values greater than 50 and still had excellent UV protection after 10 washes. According to the ASTM D1230 standard, the flame test at a 45° angle, the total burn time and residual mass retention of the fabric sample after combustion was higher than that of the untreated fabric and retained nearly 70% of the performance after 10 washes. The fabric samples had bacteriostatic zones of 18 mm and 15 mm diameter for *Staphylococcus aureus* and *Escherichia coli*, respectively, showing good antibacterial properties. Synthesis and characterization of nano-ZnO particles known for high absorption of UV radiation was reported by Farouk et al. [154]. Modified nano-ZnO particles with sol-gel based inorganic-organic hybrid polymers were applied to cellulosic cotton and cotton/polyester fabrics based on GPTMS. For the development of highly UV-protecting textiles, the use of hybrid polymers modified with ZnO could be one approach. The inorganic UV-absorber ZnO is substantially stable against degradation and it is non-toxic. Rilda et al. [155] prepared TiO_2_-SiO_2_ powders by a sol-gel method to provide cotton fabric with a uniform coating by using a spin coater. The results showed that the TiO_2_-SiO_2_-coated cotton had a ratio of Ti:Si of 1:2, which achieved the best self-cleaning effect in the degradation of methylene blue. Wang et al. [156] treated cotton fabrics with TiO_2_ hydrosols for multifunctional properties with butyl titanate (IV) as precursor. The photocatalytic activity of TiO_2_ prepared by low temperature steaming method is higher than that of TiO_2_ dried at 150 °C. Studies have shown that the fabric samples have excellent durability and self-cleaning properties. Shaban et al. [157] used a sol-gel method to prepare ZnO nanoparticles and applied them on cotton fabric to study the self-cleaning effect. The results show that the ZnO nanoparticles have high transition energy and photocatalytic activity under ultraviolet irradiation. For cotton fabric coated with ZnO nanoparticles or ZnO solution, methyl orange dye can be degraded under sunlight (7 h irradiation decomposition rate of 73%) and 200 W light (7 h irradiation, decomposition rate 26.4%).

### 2.5. Antibacterial Finishes

By applying the sol-gel method for antibacterial finishing, there has been positive outcome of eco-friendly, less utility of chemical, less temperature treatment, minimal toxicity to human health, based on the inherent properties of textile materials, and long-lasting antibacterial properties of the finished fabrics. It is the known that varied types of sol-gel systems have bacteriostatic or antibacterial effects. Successful antibacterial finishing by sol-gel process can be obtained by the anatase-modified TiO_2_ and along with various metals such as silver, copper, zinc and nickel. Poli et al. [158] prepared a Zn-based sol which was mixed with a hybrid precursor such as GPTMS to produce the Zn-containing silica coatings on cotton fabrics to obtain antibacterial activity (*Staphylococcus aureus* and *Klebsiella pneumoniae*).

**Figure 22:**
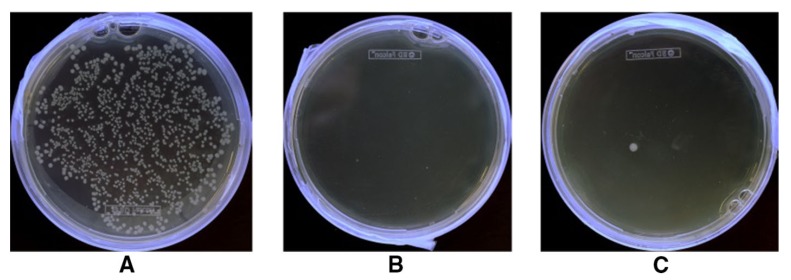
(**A**) Zone of inhibition (*E. coli*) on the number of colony-forming unit (CFU) after 24 h. untreated sample; (**B**) 3% addition of Zinc acetate and (**C**) 3% addition of Zinc acetate-GPTMS (Reproduced from [158], with kind permission of Elsevier).

**Figure 23:**
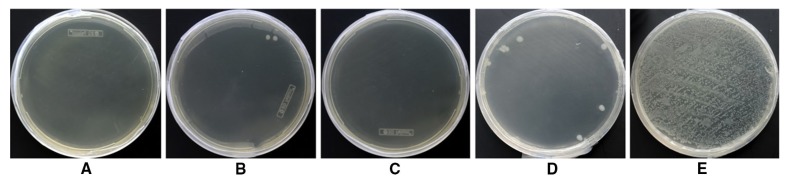
(**A**) Zone of inhibition (*E. coli*) on the 3% Zinc acetate on the number of CFU after 24 h. unwashed sample; (**B**) after 1 washing cycle; (**C**) 5 washing; (**D**) 10 washing cycles and (**E**) 20 washing cycles (Reproduced from [158], with kind permission of Elsevier).

Figure 22 shows the zone of inhibition of Zn-GPTMS-incorporated fabric which provides a better antibacterial activity against Gram-negative bacteria. On the other hand, uncoated fabric shows a rapid growth of the bacterial activity. Figure 23 shows ZnAC and GPTMS-ZnAc solution-treated fabrics and their bacterial growth activity was extremely reduced as compared to the untreated samples. These effects can sustain for up to 10 washing cycles and small colonies are visible after 10 washing cycles. Nevertheless, after 20 washings, visible bacterial colonies can be found. Mahltig et al. [96] reported a polyamide fabric with an antibacterial finish with high washability. An antimicrobial-modified SiO_2_ sol containing a silver component was applied on polyamide fabric. The antimicrobial properties of the coated polyamide fabric were determined for the bacterium *Escherichia coli*, and significant antimicrobial effects were observed even after 40 washes. In order to apply antibacterial materials using a sol-gel method, Jun et al. [159] prepared silver-doped silica thin films. The initial solution was manufactured from 1:0.24:3.75:2.2 molar ratios of Si(OC_2_H_5_)_4_): AgNO_3_:H_2_O: C_2_H_5_OCH_2_CH_2_OH and after adjusting with 0.5 N HNO_3_ solution the pH value was 3. Infrared (IR) spectroscopy, ultraviolet-visible, scanning electron microscopy and X-ray diffraction were used to examine the formation of silver-doped glassy silica thin films under varied temperatures. These analysis data reveal that the silver ions were fully captured in the silica matrix and at 600 °C annealing temperature it can achieve their reduction. The film attachment method results in antibacterial effects of silica thin films against *Escherichia coli* and *Staphylococcus aureus*. The coated films had amazing antibacterial properties.

### 2.6. Wrinkle-Resistance Finishing

Silane coupling agents have been used to produce textile fabrics with better wrinkle-free properties without requiring the addition of formaldehyde. This method also does not affect the base properties of textile fabrics (whiteness and strength loss) [160,161]. Schramm et al. [162] treated cotton fabrics with metal alkoxides (aluminum isopropoxide (AIP), titanium tetraisopropoxide and zirconium tetrabutoxide (ZTB)) or hydrophobic trialkoxysilanes (methyltriethoxysilane (MTEOS), OTES together with nanosols containing GPTMS. The wrinkle resistance of the fabric treated with the metal alkoxide alone did not improve, while the fabric treated with GPTMS had wrinkle resistance. Addition of AIP and OTES or MTEOS to the GPTMS solution increased the dry wrinkle recovery angle of the finished fabric from 263° to 289°, 290° and 294°, respectively. The tensile properties of the fabric treated with the GPTMS solution were reduced by 10% compared to the untreated fabric. The addition of the AIP solution to the GPTMS solution resulted in a further loss of 10% in tensile strength, while the addition of OTES and MTEOS solutions to the solution containing GPTMS and AIP increased the tensile strength of the treated fabric by 10%. When the fabric was treated with a GPTMS solution containing AIP or AIP and Dynasylan F8815 (a fluorinated alkyl functional water-based oligosiloxane), MTEOS and OTES, the contact angle values were increased from hydrophilicity to 112, 147, 145 and 136º, respectively. They also found that BTCA pretreatment improved the wrinkle resistance of fabrics treated with GPTMS solutions but reduced the tensile strength of fabric samples.

### 2.7. Other Functional Finishing

#### 2.7.1. Anti-Felting Finishing on Wool

Three decades ago Guise et al. [163] studied the shrink-proofing resistant finishing of wool with 3(2-aminoethylamino)-propyltrimethoxysilane with different alkoxy or amino functional groups and PDMS-diols. The two amino side group-containing trialkoxysilane provides better shrink proofing, however, the amino and alkoxy group had a significant influence on the shrink proofing finish. Shen et al. [164] produced the durable shrink-resist wool with silane containing 3-mercaptopropyl trimethoxysilane (MPTMS). In a sol-gel hybrid, epoxy or mercapto groups were added to enhance the durability. When mixing of an enzyme treatment and sol-gel polymer coatings occurs, the capacity to produce lighter and softer knitted wool fabrics is possible together with durable shrink-resistance and overwhelming whiteness. When the wool is pre-treated with sodium sulphite, disulphide bonds are broken in the cystine linkages on wool to form thiol groups, therefore resulting in an upgrading of the bonding reaction between the thiol groups in MPTMS with the wool. The hybrid sol-gel network can be re-established by cross-linking within wool fiber. Additionally, compared to MPTMS coupling group within the hybrid sol-gel yields great performance than that of GPTMS when considering the following terms of polymer uptake, fabric shrink resistance, whiteness and durability to washing.

**Scheme 1:**
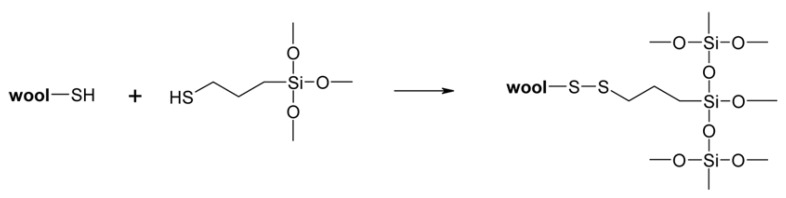
The possible reaction of MPTMS with wool (Reproduced from [164], with kind permission of Springer).

**Figure 24:**
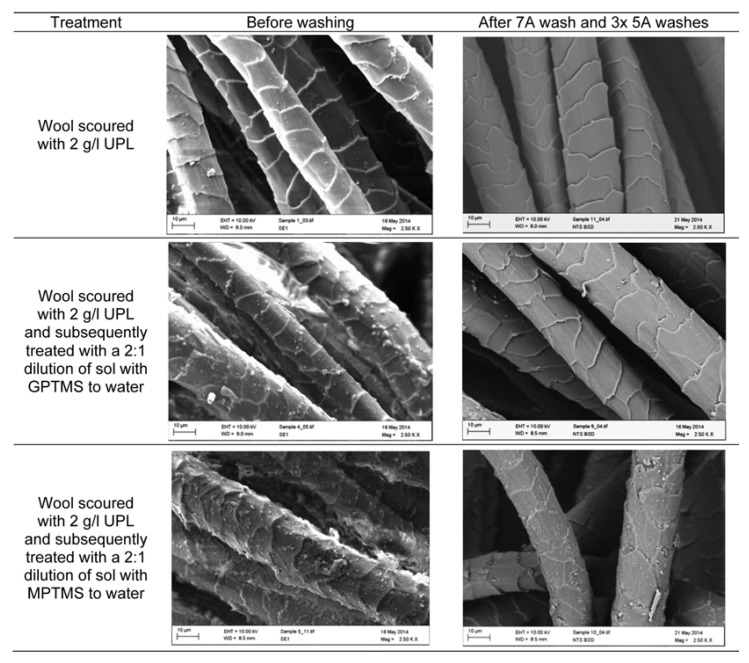
SEM images of wool scoured with 2 g/L UPL then followed by treatment with a 2:1 dilution of sol-gel containing either GPTMS or MPTMS: before washing and after 7A and 3 × 5A washes (Reproduced from [164], with kind permission of Springer).

The chemical reaction between MPTMS and wool is shown in Scheme 1. From the SEM results (Figure 24), after several washes the sol-gel coated wool fabric shows significant improvements. The control fabric shows 16.61% shrinkage after 3 × 5A washes. On other hand the sol-gel coated samples show 7.31%, which means almost 50% reduction of shrinkage after sol-gel coating. However, the results vary with respect to the type of silane used, GPTMS was washed out after 3 × 5A washes (see Figure 24). The bonds between wool and GPTMS are weak in the lipid layer, which significantly decreases the durability. On the other hand, the MPTMS was not washed off with respective washing without affecting the whiteness value of the coated wool fabric.

#### 2.7.2. Heat Storage Temperature Adjustment

Chen et al. [165] developed a thermal storage composite. For this phase change materials were incorporated into the composite with the help of a sol-gel coating method. The results show better thermal storage and the material is suitable for commercial and residential buildings, providing better thermal storage.

#### 2.7.3. Photochromic/Thermochromic Color Change Finishing

**Figure 25:**
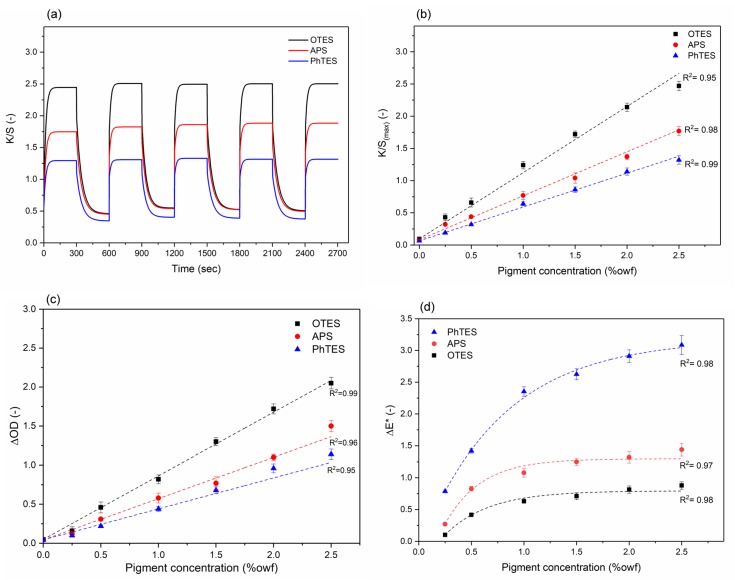
K/S values of PET fabric: (**a**) Photochromic pigment-2.50 wt.%; (**b**) K/S_(max)_; (**c**) ΔOD and (**d**) ΔE* values (Applied permission from [170], with kind permission of Taylor and Francis).

Cheng et al. [166,167,168] prepared a photochromic wool fabric by simple sol-gel coating and the photochromic response from the sol-gel coating provides a fast kinetic response. In addition, the sol-gel improves the abrasion and washing durability of photochromic dyes. However, the silane- containing rigid structure affects the fabric handling properties [167,169,170]. Additionally, the silane chemistry and its network affects the photochromic response which was extensively studied by Pardo et al. [171,172,173,174,175]. Periyasamy et al. [170] studied the effect of the photochromic response on sol-gel coated polyester fabrics. The color buildup for the sol-gel coated fabric is dependent on the type and chemical structure of the silanes used. Usually, a flexible aliphatic structure containing silanes provides better color build-up than a silane containing rigid aromatic structures. The rigid aromatic structures reduce the absorption of photochromic dyes under UV radiation followed by a reduced photochromic response. Therefore, the photochromic response is purely dependent on the chemistry, size and shape and space of the pores of the silica network. The color strength values (K/S), K/S_(max)_, changing in optical density and color difference (dE*) values of silane coated photochromic fabric is shown in Figure 25. 

In another work Parhizkar et al. [176,177] prepared a photochromic textile fabric with the help of sol-gel coating with various silanes and the photochromic response depended on the type of silane used.

#### 2.7.4. Improving the Durability/ Wear Resistance of Fabrics

Brzeziński et al. [178] prepared hybrid SiO_2_/Al_2_O_3_ sols and applied them to textile fabrics to protect against abrasion. Hybrid SiO_2_/Al_2_O_3_ sols were synthesized from (3-glycidoxypropyl) trimethoxysilane and aluminum isopropoxide. The overall results show that this treatment increased the abrasion resistance up to five times. 

**Figure 26:**
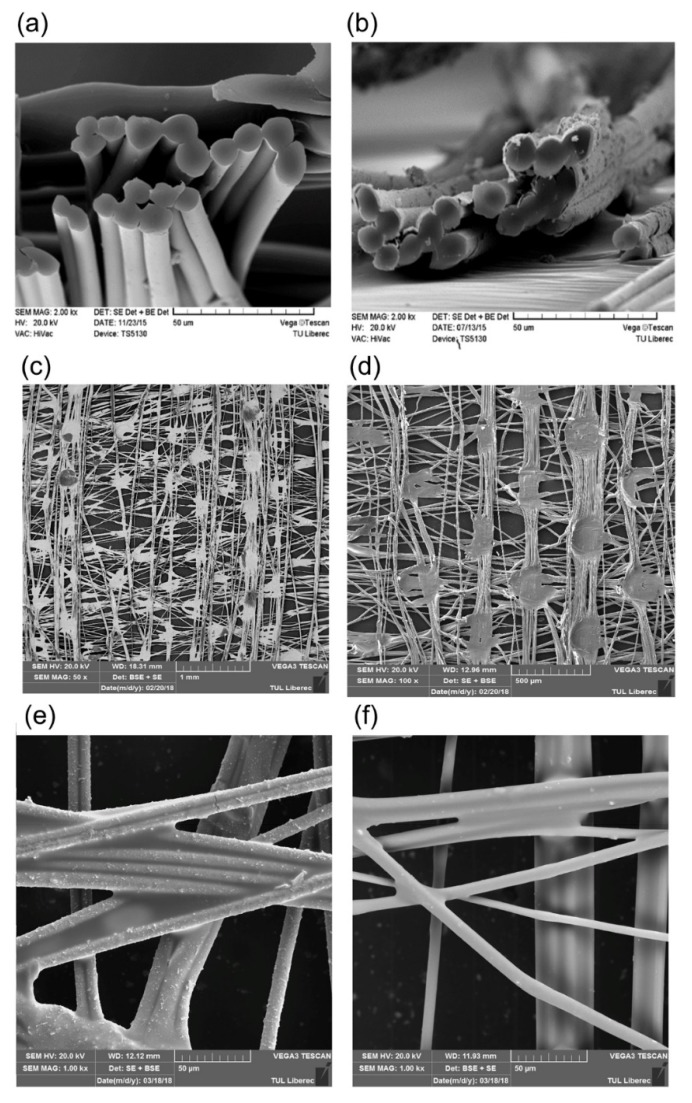
(**a**) Cross section views of nonmetal coated fabric; (**b**) metal coated fabric; (**c**,**e**) before sol-gel coating; (**d**,**f**) after sol-gel (PhTES) coating (Reproduced from [179], with kind permission of Elsevier).

Brzeziński et al. [75] improved the wear resistance of polyester-cotton blend fabric by using simple sol-gel coating techniques, where GPTMS and aluminium isopropoxide (AIP) were used as precursors. After coating, the abrasion resistance increased and it prevented the subsequent formation of pilling. In addition, authors improved the wear resistance of polyester/cotton fabrics by sol-gel coating with nanoparticles [75]. Periyasamy et al. [179,180,181,182] studied the effect of sol-gel coating on electromagnetic interference (EMI) shielding for metal-coated nonwoven fabric. The main motive of this work to stabilize the metal (copper) on the fabric surface, since copper has lower affinity towards the fabric. Sol-gel treatments are very simple processes which can stabilize metal particles on the fabric surface which increases the durability, as shown in Figure 26f. From the images it is confirmed that the Si-O-Si sols are distributed uniformly and form a thin film on the surface of metal-coated fabric which helps stabilize the metal particles on the fabric surface.

**Figure 27:**
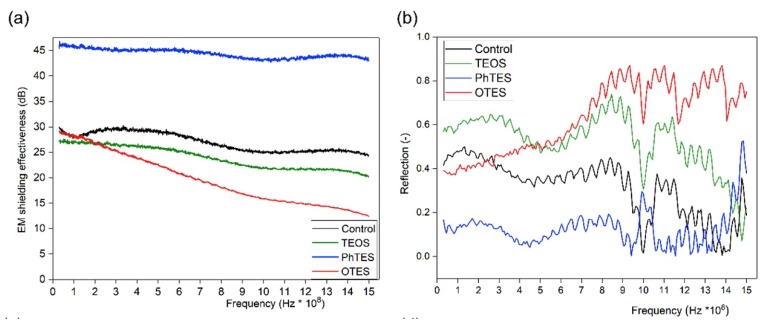
(**a**) EM shielding effectiveness of silanized metal coated fabric and (**b**) reflection effectiveness (Reproduced from [179], with kind permission of Elsevier).

Figure 27 shows the impact of sol-gel coating on EMI shielding and reflectance effectiveness for the metal-coated ultralight fabric. The electromagnetic shielding effectiveness (EMSE) values are varied with respect to the type of silane used, out of which phenyltrimethoxysilane (PhTES)-coated copper-coated fabric shows increased EMSE values (43.11 dB) compared to the control (i.e. copper coated without sol-gel coated) fabric (27.22 dB). This is due to the chemical structure of PhTES and its aromatic rings which could absorb the electromagnetic energy. In addition, it reduces the reflection effectiveness (Figure 27b). Contrarily, silanes which have aliphatic chains show reduced EMSE values.

### 2.8. Multifunctional Finishing

Onar et al. [183] prepared multifunctional (waterproof, oil-repellent and flame-retardant) cotton fabrics by a sol-gel coating technique. TEOS and ethanol were used prepare the nanosol, then acids are added to allow its acidic hydrolysis. Later guanidine dihydrogen phosphate and hexadecyl-trimethoxysilane were mixed into the above nanosol. The cotton fabric was padded and dried at 100 °C and cured at 160 °C for 1 min. Fabric treated with nanosol has better washing durability in terms of flame retardancy as well as water-oil-repellence properties. Pan et al. [39] prepared a superhydrophobic (water contact angle of 146.27°) and UV-blocking (UPF value of 164.06) cotton fabric by a sol-gel method. Memon et al. [184] treated polyester fabrics with TiO_2_-doped SiO_2_ nanosol. It has been observed that the crease resistance and UV protection properties were improved, while the gas permeability and whiteness index values were slightly decreased. Simoncic et al. [185] used a one-two-step method for multifunctional sol-gel finishing of cotton fabrics. The one-step process was found to be more effective in producing water-oil-repellent coatings, while the two-step process provided higher antimicrobial activity and better wash fastness. Mahltig et al. [186] applied sol-gel coatings to viscose and polyamide textiles to obtain both waterproof and antibacterial and found that the comfort of the fabric was not adversely affected.

Textor et al. [38] prepared textiles with water repellence and antistatic properties with a sol-gel- based surface treatment. For hydrophobic treatment, OTES was used to hydrolyze under alkali conditions with alkyltrialkoxysilanes, in addition, they added aminoalkyltrialkoxysilanes for better hydrophobic properties. The results show that treated fabric was able to repel water and absorb water vapor to improve the antistatic effects.

Shafei et al. [187] developed eco-friendly flame retardant and antibacterial finishings on cotton fabrics with a sol-gel coating. For this finishing the authors used chitosan phosphate, BTCA and TiO_2_ nanoparticles. TiO_2_ nanoparticles are prepared by a sol-gel preparation technique, and the resulting fabric shows better flame retardant and antibacterial properties. Utilization of BTCA increases the stability of chitosan phosphate and TiO_2_ nanoparticles on cotton fabric.

Rana et al. [188] studied SiO_2_ nano-gels with dimethyldimethoxysilane (DMDMS) and TEOS as precursors along with silver nitrate, potassium bromide and titanium tetrachloride. The synthesized AgBr-TiO_2_ nanoparticles are treated by a spraying process to enhance the water repellency, ultraviolet protection and antibacterial properties of the cotton fabric. The fabrics were treated with SiO_2_ nanosols containing AgBr-TiO_2_ particles, producing the contact angles of the fabrics before washing were 145.8°. Nevertheless, the fabric treated with SiO_2_ nanogel/AgBr-TiO_2_ particles had excellent UPF value of 41.9, the antibacterial activity was good, and the inhibition rate against Escherichia coli is 99.88%.

Naggar et al. [189] indicated that the cotton fabric treated by the TiO_2_ nanosol in situ and then treated by the two-step urea nitrate solution has excellent antibacterial properties, and the inhibition rates against *Staphylococcus aureus* and *Escherichia coli* are respectively 99.4% and 99.37% with excellent UV protection and good washing resistance.

Sivakumar et al. [190] studied the antibacterial, UV-blocking, detergency and self-cleaning properties of cotton/polyester fabrics with TiO_2_ nanoparticles using titanium tetraisopropoxide as a precursor, followed by 3-aminopropyltriethylsilane (APS). The oxysilane and the silicone oil are finished in a rolling baking process. The fabric treated with the modified TiO_2_ displyed the maximum photocatalytic activity and antibacterial activity, while the fabric treated with the unmodified TiO_2_ obtained a maximum UPF value.

Behzadnia et al. [191] developed a photochemical synthesis of N-Ag/TiO_2_ on wool fabrics using titanium isopropoxide and silver nitrate as precursors and ammonia as nitrogen dopant. The effects of pH, ultrasonic treatment and precursor concentration on the antibacterial and photocatalytic properties of wool fabrics were investigated. The N-TiO_2_ nanocomposite doped with Ag on wool improves the self-cleaning, photocatalytic and antibacterial properties of N-doped TiO_2_ nanocomposites on wool, while the ultrasonically treated samples containing N-Ag/TiO_2_ have higher self-cleaning and photocatalytic properties.

Gu et al. [192] demonstrated the flame retardancy and water repellency of cotton fabrics treated with phosphorus-doped HDTMS hydrosol. It was found that the LOI values, the fabric residual ratio at 600 °C, and the fabric contact angle increased from 18.5, 13.3%, and 0° to 29.4, 29.7%, and 134.6°, respectively, compared to the untreated fabric.

Hao et al. [193] studied polymer nanocomposites (PNs) synthesized by the in-situ sol-gel method to achieve multi-functional finishing of cotton fabrics. The crosslinked polysiloxane (CLPS) and APTES treated by an in-situ sol-gel process can form the silica sol (CLPS-SiO_2_). APTES-CLPS or CLPS-SiO_2_ could be dissolved in redistilled ethyl acetate to form a solution, and later the cotton fabric can be soaked in the above solutions for few seconds and immediately padded with 80% wet pickup. Then the treated fabric is dried at 100 °C for 5 min and cured at 170 °C for 2 min to stabilize the sol-gel coating. The results of fabrics treated with CLPS-SiO_2_ have lower mass loss in thermogravimetric (TGA) analysis and better thermal stability. Due to the presence of SiO_2_ nanoparticles, the square root roughness value (4.528 nm) was higher than that of fabric samples treated with APTES-CLPS, the CA of the fabric reaches 158°, and the superhydrophobic property is lost after 10 washing cycles.

## 3. Future Trends and Challenges of Sol-Gel Finishing in Textiles

Over a past few decades, the development of innovative multi-disciplinary approaches in textile research and development has brought about unceasing functional changes in the textile and clothing industry. Sol-gel based textile finishing is one among them. The thin-coat finishing of textiles carried out by the sol-gel methods is gaining greater and greater importance owing to its suitability for the versatile functionalization of textiles to impart properties that are difficult and even impossible to obtain with the use of conventional finishing methods. Since the 1960s, sol-gel coating methods for substrates such as metals, glass, and ceramics have been extensively studied. In the past few decades, research on sol-gel technology has focused on making the functionality of textile materials an alternative to conventional textile finishing. The sol-gel technology can improve the water and oil repellency, flame retardancy, UV resistance, antibacterial property and anti-wrinkle properties of textiles, and the method has the characteristics of simple process, excellent functionality, environmental friendliness and long-lasting functionality.

Globally the textile market is mainly dependent on the revolution in high-performance textile products, among them one of the prominent driving forces is sol-gel assisted textile finishing, allowing high-performance textiles to stay ahead of the competition. Market volatility and world-wide competition are the two major factors influencing the textile industry. Henceforth, there arises an urge to boost the ability to produce and merchandize supreme quality and in enriching the products. Even though there are optimistic characteristics features in sol-gel based textile finishing, there are still a lot of items that should be considered. The foremost issue is cost, which has the capacity to limit the expansion of sol-gel based coatings on textiles and mass production. Stated differently, sol-gel coating methods provide functional finished products that seem to be overpriced and require R&D spending in the textile industry. In spite of the fact the long lasting properties of the products finished using these methods are noteworthy, in order to achieve the goal of reaching the common man as well as to a particular community it is expected that the forthcoming technology on textile finishing process must be inexpensive. As a result of the enormous economic potential, besides scientists and researchers, businesses are also attracted to the unique and new properties of sol-gel materials.
